# A high-fat meal impairs muscle vasodilatation response to mental stress in humans with Glu27 β_2_-adrenoceptor polymorphism

**DOI:** 10.1186/1476-511X-9-55

**Published:** 2010-06-02

**Authors:** Marcia MG Gowdak, Mateus C Laterza, Maria Urbana PB Rondon, Ivani C Trombetta, Alexandre C Pereira, José Eduardo Krieger, Carlos Eduardo Negrão

**Affiliations:** 1 Unit of Cardiovascular Rehabilitation and Exercise Physiology, Heart Institute (InCor), University of São Paulo Medical School, São Paulo, Brazil; 2 School of Physical Education and Sports, University of São Paulo, São Paulo, Brazil

## Abstract

**Background:**

Forearm blood flow responses during mental stress are greater in individuals homozygous for the Glu27 allele. A high-fat meal is associated with impaired endothelium-dependent dilatation. We investigated the impact of high-fat ingestion on the muscle vasodilatory responses during mental stress in individuals with the Glu27 allele and those with the Gln27 allele of the β_2_-adrenoceptor gene.

**Methods:**

A total of 162 preselected individuals were genotyped for the Glu27Gln β_2_-adrenoceptor polymorphism. Twenty-four individuals participated in the study. Fourteen were homozygous for the Gln27 allele (Gln27Gln, 40 ± 2 years; 64 ± 2 kg), and 10 were homozygous for the Glu27 allele (Glu27Glu, 40 ± 3 years; 65 ± 3 kg). Forearm blood flow was evaluated by venous occlusion plethysmography before and after ingestion of 62 g of fat.

**Results:**

The high-fat meal caused no changes in baseline forearm vascular conductance (FVC, 2.2 ± 0.1 vs. 2.4 ± 0.2; *P *= 0.27, respectively), but reduced FVC responses to mental stress (1.5 ± 0.2 vs. 0.8 ± 0.2 units; *P *= 0.04). When volunteers were divided according to their genotypes, baseline FVC was not different between groups (Glu27Glu = 2.4 ± 0.1 vs. Gln27Gln = 2.1 ± 0.1 units; *P *= 0.08), but it was significantly greater in Glu27Glu individuals during mental stress (1.9 ± 0.4 vs. 1.0 ± 0.3 units; *P *= 0.04). High-fat intake eliminated the difference in FVC responses between Glu27Glu and Gln27Gln individuals (FVC, 1.3 ± 0.4 vs. 1.2 ± 0.4; *P *= 0.66, respectively).

**Conclusion:**

These findings demonstrate that a high-fat meal impairs muscle vasodilatation responses to mental stress in humans. However, this reduction can be attributed to the presence of the homozygous Glu27 allele of the β_2_-adrenoceptor gene.

## Background

Previous studies have demonstrated that a meal containing high levels of fat causes postprandial hypertriglyceridemia. The elevation of plasmatic triglyceride levels reduces LDL-cholesterol size, changing its distribution to a smaller and denser LDL-cholesterol population. The consequence of this alteration in LDL-cholesterol is its conversion from a reduced form to an oxidized form [1]. The oxidized LDL-cholesterol has been associated with impaired endothelium-dependent dilatation, which seems to be mediated by enhancement in oxygen-free radical production and a reduction in nitric oxide synthesis [2].

Accumulated evidence shows that muscle vasodilator response during physiological maneuvers depends, in great proportion, on the release of nitric oxide from endothelial cells [3-5]. In addition, in humans, the endothelial production of nitric oxide during mental stress and exercise is mediated by β_2_-adrenergic-receptor stimulation [6,7]. Brachial intra-arterial infusion of propranolol significantly reduces forearm blood flow responses during mental stress and exercise in healthy individuals [7]. Recent investigations demonstrate that a polymorphism of the N-terminus β_2_-adrenoceptors caused by exchange of an amino acid at position 27 (Glu for Gln) increases vascular responses in humans [8-10]. Cockroft et al [8] reported that forearm vasodilator responses to intra-arterial infusion of isoproterenol were greater in individuals who were homozygous for the Glu27 allele than in those who were homozygous for the Gln27 allele of the β_2_-adrenoceptor. Trombetta et al [7] reported augmented muscle vasodilator responsiveness to mental stress and handgrip exercise in women who were homozygous for the Glu27/Gly16 haplotype of the β_2_-adrenoceptor gene. These observations have been recently replicated in children with the Glu27 allele of the β_2_-adrenoceptor gene [11]. These findings suggest that the augmented muscle blood flow during physiological maneuvers in individuals with the Glu27 allele is due to a greater nitric oxide bioavailability. Whether fat ingestion reduces nitric oxide production, it is reasonable to expect that the impairment in muscle blood flow responses caused by a high-fat meal will be greater in humans who are homozygous for the Glu27 allele than in humans who are homozygous for the Gln27 allele of the β_2_-adrenoceptor gene. In this study, we investigated the impact of high-fat ingestion on the muscle vasodilatory responses during mental stress in individuals with the Glu27 allele and those with the Gln27 allele of the β_2_-adrenoceptor gene.

Our hypothesis was that the reduction in forearm vasodilatation caused by a high-fat meal would be more marked in individuals who were homozygous for Glu27 than in those homozygous for the Gln27 allele of the β_2_-adrenoceptor gene.

## Methods

### Subjects

A total of 162 preselected individuals from the Cardiovascular Screening Outpatient Clinic [Heart Institute (InCor), University of São Paulo, Medical School] were genotyped for the Glu27Gln β_2_-adrenoceptor polymorphism. Genomic DNA was extracted, according to standard techniques, from leukocytes in samples of whole blood. The studied polymorphism was detected by polymerase chain reaction-restriction fragment length polymorphism (PCR-RFLP) as previously described [12]. Quality control for these assays was assessed by randomly selecting 50 samples to be regenotyped by 2 independent technicians.

We found Gln27/Gln27 (n = 82), Gln27/Glu27 (n = 64), and Glu27/Glu27 (n = 16) β_2_-adrenoceptor alleles. Individuals heterozygous were excluded from the protocol. Inclusion criteria were normal health status, not physically active, nonsmokers, body mass index <25 kg/m^2^, and age between 30 and 55 years (women before menopause). We randomly selected 25 Gln27Gln individuals. All 16 Glu27Glu individuals were selected for the study. The individuals were screened for cardiovascular, endocrine, and metabolic disorders. Nine subjects were excluded from the protocol for having either dyslipidemia (n = 6) or hypertension (n = 3). After preliminary examination, the individuals were invited to participate in the protocol. Seven individuals did not want to participate in the study, and one volunteer panicked during the mental stress protocol. Thus 14 individuals homozygous for the Gln27 allele (Gln27 group) and 10 homozygous for the Glu27 allele of the β_2_-adrenoceptor polymorphism (Glu27 group) were included in the study. The individuals took no medication or vitamins for 3 months before the study and were told to abstain from exercise 2 months before the study. They were also asked not to use caffeine and alcohol one day before the study. All women were studied between the first and the fifth day after the onset of menstruation.

The Institutional Ethics Committee approved the protocol (ref.:272/04), and all volunteers provided a written informed consent.

### Measurements and Procedures

#### Forearm Blood Flow

Forearm blood flow was measured by venous occlusion plethysmography [13]. The nondominant arm was elevated above heart level to ensure adequate venous drainage. A mercury-filled silastic tube attached to a low-pressure transducer was placed around the forearm and connected to a plethysmograph (Hokanson, Bellevue, WA). Sphygmomanometer cuffs were placed around the wrist and upper arm. At 15-s intervals, the upper cuff was inflated above venous pressure for 7-8s. Forearm blood flow (mL/min/100 mL) was determined based on a minimum of 4 separate readings. Forearm vascular conductance was calculated by dividing forearm blood flow by mean arterial pressure. The reproducibility of forearm blood flow measured at different time intervals in the same individual expressed as milliliters per minute per 100 milliliter tissue in our laboratory is r = 0.93 [7].

#### Blood Pressure and Heart Rate

Blood pressure was monitored noninvasively and intermittently from an automatic and oscillometric cuff (Dixtal, DX 2710; Manaus, Brazil) [14,15]. The cuff was inflated every 30s. Heart rate was monitored continuously through ECG lead II.

#### Mental Stress Testing

Mental stress was elicited by the Stroop color-word test [16]. During this test, subjects were shown a series of names of colors written in different colored ink from the color specified. The subjects were asked to identify the color of the ink, not to read the word.

#### Laboratory Tests

Total serum cholesterol levels, HDL-cholesterol, and triglycerides were measured with the automated Cobas MIRA system (F. Hoffmann-La Roche, Switzerland), and LDL-cholesterol was calculated by the simplified equation suggested by Friedewald [17]. Plasma glucose level was determined by a commercial enzymatic method (Cobas-Roche, Germany) and insulin by immunofluorometric assay (AutoDelfia, Turke, Finland).

### Experimental Protocol

After patients had a good night's sleep, a fasting blood sample was drawn for serum total-, LDL-, and HDL-cholesterol, triglycerides, glucose, and insulin determination. All studies were performed in a quiet temperature-controlled (21°C) room at approximately 7:30 A.M. The arm was positioned for venous plethysmography, and the individual rested quietly for 15 minutes. Baseline forearm blood flow, blood pressure, and heart rate were recorded for 3 minutes. Mental stress was then performed for 3 minutes. Forearm blood flow, blood pressure, and heart rate were recorded continuously during mental stress. The task difficulty was determined on completion of the protocol by using a standard 5-point scale: 0, Not stressful; 1, Somewhat stressful; 2, Stressful; 3, Very stressful; and 4, Very very stressful. The protocol was repeated before and 3 hours after patients had ingested a high-fat meal that consisted of a milkshake with 924 calories, 62 g of fat, 40 g of saturated fat, and 200 mg of cholesterol. Lipid, insulin, and glucose determinations were also repeated after the meal.

### Statistical Analysis

Data are presented as mean ± SEM. The paired Student *t *test was used to compare data before and after the high-fat meal. Baseline measurements between individuals with Gln27Gln and Glu27Glu of the β_2_-adrenoceptor gene were compared using the unpaired Student *t *test. The responses of forearm blood flow, forearm vascular conductance, mean blood pressure, and heart rate were analyzed by the 2-way ANOVA with repeated measures. When statistical significance was found, Scheffé's post-hoc comparisons were performed. Probability values of <0.05 were considered statistically significant.

## Results

### Impact of High-Fat Meal

**Figure 1:**
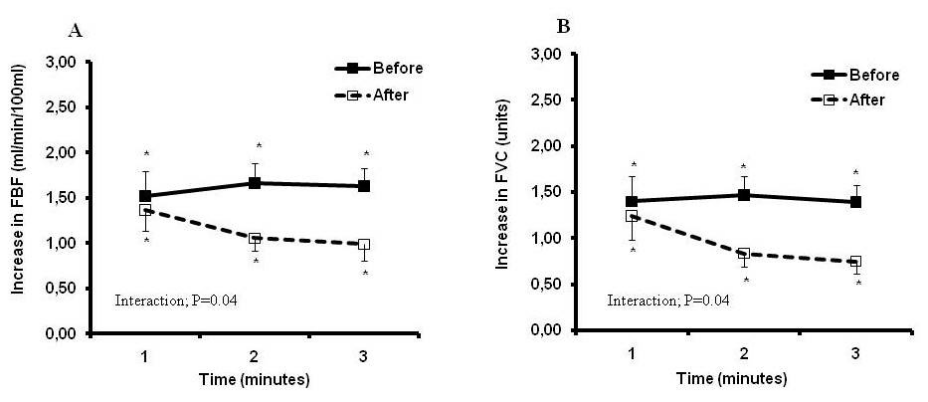
**Forearm blood flow (FBF, Panel A), forearm vascular conductance (FVC, Panel B) responses (absolute change) during mental stress before and after high-fat ingestion in the total group of subjects**. * = from baseline, † = after meal difference *P *< 0.05.

The effects of a high-fat meal on metabolic and hemodynamic parameters are shown in Table 1. The high-fat meal significantly increased total cholesterol and triglyceride levels, but caused no changes in LDL- and HDL-cholesterol levels. The high-fat meal did not change insulin levels, but significantly reduced glucose levels. Baseline forearm blood flow and forearm vascular conductance were unchanged by ingestion of the high-fat meal. Baseline mean blood pressure levels were lower and heart rate greater after the high-fat meal. During mental stress, the high-fat meal significantly reduced forearm blood flow (Figure 1A, Interaction, *P *= 0.04) and forearm vascular conductance responses (Figure 1B, Interaction, *P *= 0.04). Mean blood pressure responses during mental stress were unchanged by the high-fat meal (MBP, 9 ± 1 vs. 8 ± 2 mmHg; *P *= 0.38). Heart rate responses during the mental stress test were lower after the high-fat meal (HR, 12 ± 1 vs. 7 ± 1 bpm; *P *= 0.05).

**Table 1:** Metabolic and hemodynamic measurements before and after a high-fat meal.

Variable	Before (n = 24)	After (n = 24)	*P*
***Metabolic Measurements***			
Total-cholesterol (mg/dL)	179 ± 7	189 ± 7*	<0.001
LDL-cholesterol (mg/dL)	107 ± 6	100 ± 6	0.06
HDL-cholesterol (mg/dL)	52 ± 3	52 ± 3	0.43
Triglycerides (mg/dL)	100 ± 9	210 ± 20*	<0.001
Glucose (mg/dL)	88 ± 1	82 ± 2*	0.002
Insulin (μU/mL)	6 ± 1	8 ± 1	0.06
***Hemodynamic Measurements***			
Mean Blood Pressure (mm Hg)	95 ± 2	90 ± 2*	0.001
Heart Rate (bpm)	67 ± 2	72 ± 2*	0.003
Forearm Blood Flow (mL/min/100 mL)	2.1 ± 0.1	2.1 ± 0.2	0.75
Forearm Vascular Conductance (units)	2.2 ± 0.1	2.4 ± 0.2	0.27

Values are mean ± SEM. *Significant difference vs. baseline, *P *< 0.05.

### Impact of the High-Fat Meal on Gln27Gln and Glu27Glu Individuals

There were no significant differences in age (41 ± 3 vs. 40 ± 2 years, *P *= 0.96) weight (66 ± 3 vs. 64 ± 2 kg, *P *= 0.52), and BMI (23 ± 1 vs. 24 ± 1 kg/m_2_, *P *= 0.59) between Gln27Gln and Glu27Glu individuals. The effects of the high-fat meal on metabolic measures in Glu27Glu and Gln27Gln individuals are shown in Table 2. High-fat ingestion significantly and similarly increased total-cholesterol levels in both Glu27Glu and Gln27Gln individuals. The high-fat meal did not significantly change LDL- and HDL-cholesterol levels in either group. In contrast, the high-fat meal significantly and similarly increased triglyceride levels in Glu27Glu and Gln27Gln individuals. Glucose levels significantly and similarly decreased after the high-fat meal in Glu27Glu and Gln27Gln individuals. No significant changes in insulin levels were observed in the 2 study groups.

**Table 2:** Cholesterol, lipoproteins, triglycerides, glucose, and insulin levels before and after a high-fat meal in individuals with Glu27Glu and Gln27Gln of the β_2_-adrenoceptors.

Variable	Genotypes	Before	After
**Total-cholesterol (mg/dL)**	Glu27Glu	173 ± 12	188 ± 12*
	Gln27Gln	183 ± 9	190 ± 9*
**LDL-cholesterol (mg/dL)**	Glu27Glu	102 ± 10	98 ± 10
	Gln27Gln	110 ± 7	101 ± 8
**HDL-cholesterol (mg/dL)**	Glu27Glu	51 ± 3	52 ± 3
	Gln27Gln	53 ± 5	51 ± 5*
**Triglycerides (mg/dL)**	Glu27Glu	102 ± 14	212 ± 35*
	Gln27Gln	98 ± 11	209 ± 23*
**Glucose (mg/dL)**	Glu27Glu	87 ± 1	79 ± 3*
	Gln27Gln	89 ± 2	84 ± 3*
**Insulin (μU/mL)**	Glu27Glu	5 ± 1	6 ± 1
	Gln27Gln	7 ± 2	10 ± 1

Values are mean ± SEM. *Significant difference vs. baseline, *P *< 0.05.

In regard to hemodynamic parameters, no significant differences existed in baseline mean blood pressure (92 ± 3 vs. 93 ± 2 mm Hg, *P *= 0.79), heart rate (69 ± 4 vs. 66 ± 1 beat/min, *P *= 0.44), forearm blood flow (2.2 ± 0.1 vs. 2 ± 0.1 mL/min/100 mL, *P *= 0.23), and forearm vascular conductance (2.4 ± 0.1 vs. 2.1 ± 0.1 units, *P *= 0.08) between Glu27Glu and Gln27Gln individuals.

**Figure 2:**
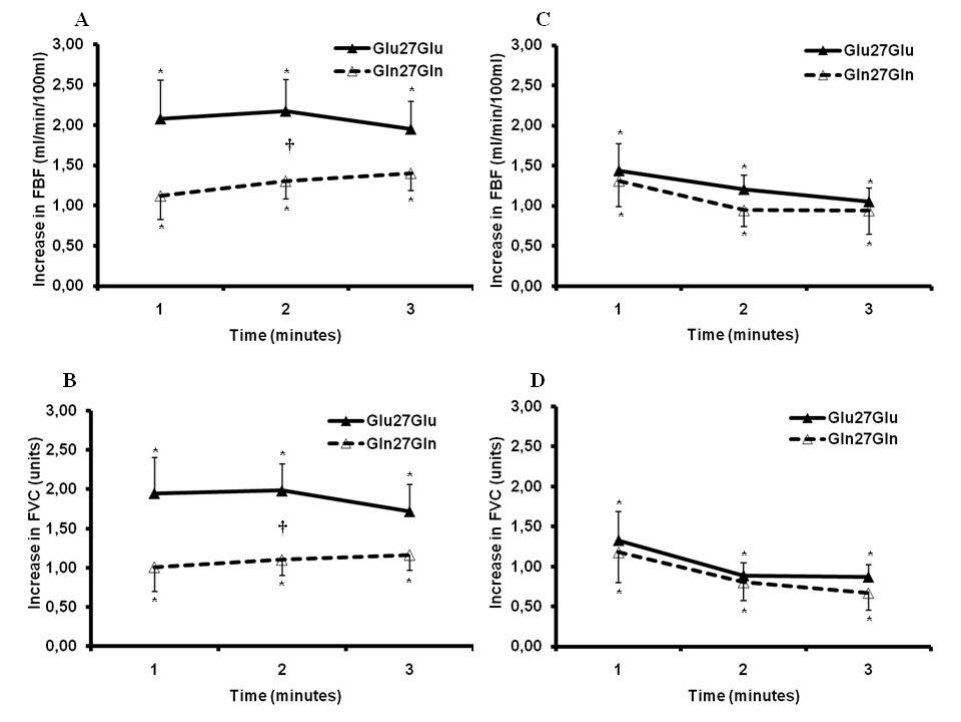
**Forearm blood flow (FBF) and forearm vascular conductance (FVC) responses (absolute change) during mental stress before (Panel A and Panel B, respectively) and after (Panel C and Panel D, respectively) high-fat ingestion in individuals with the Glu27Glu or Gln27Gln β_2_-adrenoceptor genotype**. Note that the increase in FBF and FVC in the Glu27Glu β_2_-adrenoceptor group disappears after intake of a high-fat meal. * = from baseline, † = between group difference *P *< 0.05.

Mean blood pressure and heart rate responses to mental stress are shown in Table 3. Mental stress significantly and similarly increased mean blood pressure and heart rate levels in Glu27Glu and Gln27Gln individuals. Forearm blood flow increased significantly during mental stress in Glu27Glu and Gln27Gln individuals. However, forearm blood flow responses throughout mental stress were significantly greater in Glu27Glu individuals when compared with the Gln27Gln group (Figure 2A, Group Effect, *P *= 0.05). Similarly, forearm vascular conductance levels during mental stress were significantly increased in Glu27Glu and Gln27Gln individuals, but the increase in forearm vascular conductance was significantly greater in Glu27Glu individuals (Figure 2B, Group Effect, *P *= 0.03). After the high-fat meal, no significant differences were noted in forearm blood flow and forearm vascular conductance responses between Glu27Glu individuals and Gln27Gln individuals (Figure 2C and 2D, respectively).

**Table 3:** Baseline and absolute changes in mean blood pressure and heart rate during mental stress before and after a high-fat meal in individuals with the Glu27Glu and Gln27Gln β_2_-adrenoceptor gene.

		Mental Stress							
		
		Baseline		1 min		2 min		3 min	
		
		Before	After	Before	After	Before	After	Before	After
**MBP (mm Hg)**	Glu27Glu	92 ± 3	89 ± 2	4 ± 3	7 ± 2*	7 ± 2*	10 ± 2*	9 ± 1*	8 ± 3*
	Gln27Gln	93 ± 2	90 ± 3	5 ± 2	6 ± 2*	7 ± 2*	9 ± 2*	9 ± 2*	9 ± 2*
**HR (beats/min)**	Glu27Glu	69 ± 4	73 ± 4	9 ± 2*	6 ± 3*	12 ± 2*	7 ± 2*	13 ± 2*	6 ± 3*
	Gln27Gln	66 ± 1	72 ± 2	8 ± 1*	7 ± 1*	11 ± 2*	7 ± 2*	11 ± 2*	6 ± 1*

MBP = mean blood pressure; HR = heart rate. Values are mean ± SEM. *Significant difference vs. baseline, *P *< 0.05.

The high-fat meal caused no significant changes in baseline and mental stress responses in both mean blood pressure and heart rate in Glu27Glu and Gln27Gln individuals (Table 3). The high-fat meal did not change forearm blood flow from baseline measurements in Glu27Glu and Gln27Gln individuals (before = 2.2 ± 0.1 vs. after = 2.3 ± 0.3, *P *= 0.75, and before = 2.0 ± 0.1 vs. after = 1.9 ± 0.1 mL/min/100 mL, *P *= 0.97, respectively). Similarly, the high-fat meal did not change forearm vascular conductance from baseline measurements in Glu27Glu and Gln27Gln individuals (before = 2.4 ± 0.1 vs. after = 2.6 ± 0.4, *P *= 0.56, and pre = 2.1 ± 0.1 vs. post = 2.2 ± 0.2 units, *P *= 0.45, respectively).

## Discussion

The main findings of the present study are that (1) a high-fat meal impairs reflex muscle vasodilatation during mental stress in healthy individuals; (2) the reduction in muscle vasodilatation caused by the high-fat meal is more marked in individuals who are homozygous for the Glu27Glu allele of the β_2_-adrenoceptor gene than in individuals who are homozygous for the Gln27 allele.

The defense reaction, which in humans can be elicited by a mental challenge, has been well characterized. This physiological maneuver causes an increase in cardiac output, blood pressure, and visceral vasoconstriction. All these responses work in concert to increase blood flow to skeletal muscle to prepare an animal for fight or flight [5]. More recently, it has become clear that the increase in muscle blood flow during a defensive reaction depends on local vasodilatation. β_2_-adrenoceptor stimulation provokes nitric oxide production from the endothelial cells, the consequence of which is intense muscle vasodilatation [6]. In the present study, we confirmed that mental stress increases muscle blood flow. Moreover, we provide evidence that high-fat ingestion significantly reduces skeletal muscle vasodilatation during mental stress in healthy individuals. This finding illustrates the deleterious effects of high-fat ingestion on vascular function during a regular physiological situation in daily life activities in humans. Previous studies [18,19] support the concept that high-fat ingestion increases plasma triglyceride levels, which may lead to endothelial dysfunction by lowering LDL-cholesterol particles and making them prone to oxidation [1]. The presence of oxidized products in the endothelium impairs the endothelial cell's capacity to generate nitric oxide by decreasing nitric oxide synthase activity, and hence, vasorelaxation [2]. This constellation of vascular alterations seems to play a role in the reduction of forearm blood flow responses during mental stress in the present study. In fact, the high-fat meal caused hypertriglyceridemia and forearm blood flow reduction, which suggests an association between these 2 physiological responses.

The unique observation in our study is the divergent effects of high-fat ingestion on muscle vasodilatation in individuals with the Glu27Glu β_2_-adrenoceptor gene and individuals with the Gln27Gln β_2_-adrenoceptor gene. In contrast to Gln27Gln individuals, the high-fat meal caused a significant decrease in forearm blood flow in response to mental stress in Glu27Glu individuals. The question that emerges from these findings is why individuals with the Glu27Glu genotype, who have greater vascular responsiveness during physiological maneuvers, are more sensitive to the vascular effects of high-fat ingestion. Although the definitive answer to this question is out of the scope of the present investigation, some explanations are possible for such an apparent paradox. The vascular effects of fat ingestion are greater in individuals who have more nitric oxide bioavailability, and hence, endothelial-mediated vasodilatation. But what evidence supports the increased endothelial function in Glu27Glu individuals? Recent studies have shown that forearm vasodilatory responses to intra-arterial infusion of isoproterenol are greater in individuals who are homozygous for the Glu27 allele than in those who are homozygous for the Gln27 allele of the β_2_-adrenoceptor [8]. The endothelial-mediated forearm vasodilatation during mental stress and exercise is augmented in individuals who are homozygous Glu27 for the β_2_-adrenoceptor gene when compared with individuals who are homozygous Gln27 for the β_2_-adrenoceptor gene [7-11]. In addition, the present study replicates the observation that Glu27Glu individuals have greater forearm vascular conductance in response to mental challenges than do Gln27Gln individuals. It might also be possible that the greater impact of fat ingestion on Glu27Glu individuals was mediated by metabolic alterations. The present findings do not support this hypothesis, because the changes in triglycerides caused by high-fat ingestion are the same in Glu27Glu and Gln27Gln individuals.

Despite the fact that the relative reduction in forearm blood flow responses after high-fat ingestion was more marked in Glu27Glu individuals than in Gln27Gln individuals, the absolute levels of forearm blood flow were similar in Glu27Glu and Gln27Gln individuals. Thus, individuals with the Glu27Glu genotype are not necessarily more susceptible to the deleterious effects of high-fat ingestion, but actually, they loose its augmented endothelial function by "regression to the mean." So far, the implication of such results is unknown. However, these results open new perspectives for future studies regarding the functional modulation of genetic polymorphisms of the β_2_-adrenoceptor gene.

The similarity in mean blood pressure heart rate responses after high-fat ingestion rules out the possibility that other hemodynamic adjustments beyond local blood vessel functioning explain the difference in forearm blood flow responses between Glu27Glu and Gln27Gln individuals. In addition, forearm vascular conductance, which takes into consideration mean blood pressure levels, was greater in Glu27Glu individuals. It would be legitimate to question whether the difference in forearm blood flow responses during mental challenges between Glu27Glu and Gln27Gln individuals were due to a lower level of perceived stress during color-word testing. Our data do not support this idea, because no difference was found in the perception of stress during the color-word test between groups (data not shown). Forearm blood flow was evaluated 180 minutes after fat ingestion. Thus, we have no information regarding the time course of fat ingestion effects on vascular function.

## Conclusion

In summary, the impairment in endothelial-mediated muscle vasodilatation caused by the high-fat meal is markedly influenced by the Glu27 allele of the β_2_-adrenoceptor gene. This finding contributes to the understanding of the association between the environmental condition related to high-fat ingestion present in many Western societies and the genetic regulation of vascular function mediated by β_2_-adrenoceptors.

## competing interests

The authors declare that they have no competing interests.

## Authors' contributions

MMGG participated in the design of the study, performed the statistical analysis, acquisition of data and drafted the manuscript. MCL contributed to acquisition of data and its interpretation. MUPBR and ICT contributed to the interpretation of data and helped with the statistical analysis. ACP was involved in revising the manuscript critically and contributed to its content. JEK contributed to conception and design of the study. CEN conceived of the study, participated in its design, coordination and helped to draft the manuscript. All authors read and approved the manuscript.
